# Cooperative cell–cell actin network remodeling to perform Gap junction endocytosis

**DOI:** 10.1186/s12610-023-00194-y

**Published:** 2023-08-03

**Authors:** Dominique Segretain, Mathilde Di Marco, Chloé Dufeu, Diane Carette, Alain Trubuil, Georges Pointis

**Affiliations:** 1 UMR S1147, Université Paris Descartes, 45 Rue Des Saints-Pères, 75006 Paris, France; 2 Present Address: Institut Curie, PSL Research University, CNRS, UMR144, Structure and Membrane Compartments, 75005 Paris, France; 3 Faculté de Pharmacie, Université Paris-Saclay, Saclay, France; 4 INSERM, SC10 US19 Villejuif, France; 5 MaIAGE, INRAE, Université Paris-Saclay, 78352 Jouy-en-Josas, France; 6 INSERM U 1065, Team 5 Physiopathological Control of Germ Cell Proliferation: Genomic and Non-Genomic Mechanisms, University of Nice Sophia-Antipolis, 151 Route Saint-Antoine de Ginestière BP 2 3194, 06204 Nice Cedex 3, France

**Keywords:** Annular Gap Junction (AGJ), Gap Junction (GJ), Gap Junction Plaque (GJP), Jonction gap (GJ), Plaque de jonctions gap (GJP), Jonction gap annulaire (GJA), Jonction gap (GJ), Plaque de jonctions gap (GJP), Jonction gap annulaire (GJA)

## Abstract

**Background:**

The endocytosis of Gap junction plaques (GJP) requires cytoskeletal forces to internalize such large membranous structures. Actin, which partners the connexin proteins constituting Gap junctions and is located close to Annular Gap Junctions (AGJ), could be actively involved in this physiological process.

**Results:**

Electron Microscopy and Light Microscopy images, associated with time-lapse analysis and 3D reconstruction, used at high resolution and enhanced using ImageJ based software analysis, revealed that: i) actin cables, originating from Donor cells, insert on the edge of GJP and contribute to their invagination, giving rise to AGJ, whereas actin cables on the Acceptor cell side of the plaque are not modified; ii) actin cables from the Donor cell are continuous with the actin network present over the entire GJP surface. These actin cables fuse at a single point distant from the plaque, which then detaches itself from the membrane, condensing to form an actin mass during the final internalization process; iii) the Acceptor cell participates in the last step of the endocytic invagination process by forming an annular actin structure known as an actin ring.

**Conclusions:**

Together, these data suggest that the endocytosis of GJP is an example of a unique cooperative mechanism between the Donor (the traction of its actin cables) and the Acceptor cells (forming the actin ring).

**Supplementary Information:**

The online version contains supplementary material available at 10.1186/s12610-023-00194-y.

## Résumé

**Contexte:**

L’endocytose des plaques de jonctions communicantes ou jonctions gap (GJP) nécessite les forces du cytosquelette pour internaliser ces grandes structures membranaires. L’actine, partenaire des connexines, proteins constitutives des jonctions gap (Gj), localisée proche des jonctions gap annulaires (GJA), pourrait être impliquée dans ce processus physiologique.

**Résultats:**

L’ imagerie par microscopie optique et électronique, associées avec des analyses vidéo et des reconstructions en relief/3D, examinées à haute résolution et améliorées après traitement par des logiciels développés sous ImageJ, montrent que: i) des câbles d’actine, originaires des cellules donneuses, s’insèrent sur le bord des plaques jonctionnelles et facilitent leur invagination pour former les GJA tandis que les câbles d’actine des cellules receveuses ne sont pas modifies; ii) les câbles d’actine des cellules donneuses sont en continuité avec le réseau d’actine qui couvre la totalité de la surface de la plaque. De plus, ces câbles fusionnent en un point unique, à distance de la plaque, qui se détache de la région membranaire pour former une masse d’actine à la fin du processus d’endocytose; iii) la cellule receveuse participe à l’étape ultime du processus d’endocytose de la plaque en formant un anneau d’actine.

**Conclusions:**

L’ensemble de nos résultats montrent que l’endocytose des plaques jonctionnelles est un exemple de coopération unique entre la cellule donneuse (grâce à la traction des câbles d’actine) et la cellule receveuse (anneau d’actine).

## Introduction

Gap Junction Plaques (GJP), ultrastructurally characterized by a trilaminar membrane, are formed by a hexameric arrangement of connexins (Cx) that create channels between two adjacent cells, named connexons [1]. Cell alteration and/or dysfunction implies rapid closure of connexon channels [2], which is rapidly followed by plaque endocytosis to give rise to an Annular Gap Juntion (AGJ).

Gap junction endocytosis is the final physiological fate of GJP and exaggerated GJP endocytosis could be the signature of pathological cell dysfunction, mostly of tumor progression [3, 4].

The formation, stabilization, and the subsequent internalization processes of large membranous structures such as GJP, imply initial molecular interactions with many Cx protein partners (e.g., ZO1, Dynamin, Debrin….) [5–8]. Some of these, such as tubulin and actin, are also identified as Cx partners and are directly involved in the trafficking of these structures [9, 10]. This has been supported by the results of microinjection of anti-actin Ab, or Cyto D treatment, which decrease Gap junction intercellular communication [11]. While tubulin is involved in transport of vesicles from Golgi to membranes [12], the role of actin in the trafficking of connexons and specifically in GJP endocytosis is not clearly defined.

Actin plays a physiological role in junctional structures such as adherens and tight junctions since exposure to the actin disrupting drug, Cytochalasin D, leads to alteration of these membranous structures [13]. In the primate lens, AGJ are decorated by actin filaments, suggesting that this protein is probably involved in the trafficking of these structures [14]. Other studies have revealed that actin, associated with myosin VI, participates in AGJ displacement and the accumulation of newly formed GJP [15]. The authors of this study also reported an accumulation of actin at the plaque periphery in mice myosin VI null mouse using Total Internal Reflection Fluorescence (TIRF) microscopy. By using Cytochalasin D, which induces actin depolymerization, cellular redistribution of Cx has been observed, supporting the involvement of actin in Cx trafficking and stabilization [16]. In addition, cell exposure to Ultraviolet A radiation affects actin microfilaments concomitantly with a decrease of Gj intercellular communication [17]. Together, these observations support a potential role of actin in Cx distribution and GJP formation. Other previous studies revealed that actin might also be involved in the anterograde traffic of Cx43 vesicles to the plasma membrane [18].

Previous ultrastructural analysis by electron microscopy in different cell types showed that actin localized close to GJP during the final degradation process of the AGJ [19]. More recently, it was suggested that actin/myoVI activity is required for plaque endocytosis and a schematic model for Gj endocytosis has been proposed [20]. Nanoscale analysis of endocytosis using super resolution microscopy to observe actin nucleation has reinforced the hypothesis that actin is involved in this process [21]. Directional actin forces cause membrane remodeling during progressive membrane curvature [22]. Since the endocytic process requires force to internalize the plasma membrane, the possibility that actin forces ensure GJP internalization has been strongly hypothesized.

In the testis, Gap junctions are present between Sertoli cells and between Sertoli and germ cells and play a major role during spermatogenesis [23]. By using immunofluorescence and Electron Microscopy (EM) images in Cx43-GFP transfected Sertoli cells, the present studies show the role of actin cables in GJP endocytosis. Further stereoscopic EM examination and time-lapse spinning disk fluorescence microscopy highlight the specific positioning of actin cables on the GJP periphery. The roles of the Donor and Acceptor cells, and their participation in the endocytosis of this large trilaminar membranous structure are discussed.

## Materials and methods

### Cell culture and transfection

The 42GPA9 Sertoli cell line that expresses Cx43 [24] was maintained in Dulbecco's Modified Eagle's Medium (GIBCO BRL Cergy Pontoise) containing 10% fetal calf serum and cultured at 32° C. The Cx43-GFP probe was a generous gift from M.M Falk (Department of Biological Sciences, Lehigh University, 111 Research Drive, Iacocca Hall, Bethlehem, USA). Probes were then mixed for 45 min with OptiMEM (Invitrogen SARL) and 0.75 μg of the vector was transfected using Lipofectamin (Invitrogen), according to the manufacturer's instructions and as previously described [25].

### Immunofluorescence experiments

Immunofluorescence analyses on cells were performed as described previously [24]. Cells were cultured at 32 °C on glass coverslips for 24 h and transfected with Cx43-GFP. For actin examination, a rhodamine-phalloidin stock solution was prepared according to the manufacturer’s directions (Invitrogen) and used at a dilution of 1:200 in PBS for 10 min.

Three-dimensional high resolution deconvolution microscopy analysis was performed with a wide field deconvolution microscope Nikon TE-2000E (SCM, University Paris Descartes) connected with a cooled charge-coupled device camera (Roper CoolSnap HQ2) as described previously [24]. Images were collected with NIS Element software (Nikon) and deconvoluted with AutoQuant image package algorithms.

### Time lapse analyses and spinning disk experiments

Using the deconvolution microscope, time-lapse images were collected every 10 s for 15 min or every 15 min for 140 min with NIS Element software (Nikon) and deconvoluted using AutoQuant image package algorithms. 3D video microscopy images of actin attachment to GJP were studied in Cx43-GFP transfected cells stained with rhodamine phalloidin for actin detection, using Spinning disk confocal microscopy at the Institute Curie Nikon Imaging center (Paris). Stacks of images were collected every minute.

### Ultrastructural examination

Cells were fixed with 2.5% glutaraldehyde in phosphate-buffer saline (0.1 M, pH: 7.4) for 1 h at room temperature, post-fixed in ferrocyanide-reduced osmium, dehydrated and embedded in Epon as previously reported [26]. Thin sections were counterstained with uranyl acetate and lead citrate and analyzed with a Philips CM 10 electron microscope (CNRS-UNIC, Institute A. Fessard, Gif sur Yvette, France).

### Contrasted images

High resolution Light Microscopy (LM) images, needed to accurately observe actin distribution, were contrasted with the ImageJ enhance contrast software. For Electron Microscopy (EM), images were scanned at high resolution (3200 ppp) and similarly processed. To focus on the organization of actin fibers observed on EM and LM images, their structure was manually redrawn with red lines.

### Stereoscopic imaging

For stereoscopy, EM grids were placed on the goniometric stage of the electron microscope, and stereopairs were obtained by taking pictures of the same field after tilting the specimen at − 10° and + 10° from the 0° position [27]. 3D images of the structures were then obtained, observing adjusted pairs of photographs with a stereoscope.

### Amira 3D Volumic reconstruction

In order to reconstruct entire GJP associated with actin cables, either the all-in-one Avizo™ image analysis platform or the commercial version of Amira software were used for the visualization, processing, and quantification of 3D stacks of images (ThermoFisher Scientific). The two channels of the images stacks corresponding respectively to actin staining (red channel) and connexin staining (green channel) were independently binarized using a classical thresholding algorithm and visualized together in 3D. A triangular mesh of each isosurface could also be built automatically using the Avizo software.

### Skeletonization of actin

To understand how the actin net is organized over the face of plaques, ImageJ skeletonization software was used. Using enhanced contrast images, the final 8 bit images were binarized, dilated and then the skeletonization was applied. The ImageJ Find Edges filter was applied to the skeletonized images in order to better define the organization of the actin mesh network.

### Statistical image analysis

Actin filament distribution over GJP was analyzed in cross section in EM images following contrast enhancement using ImageJ. The number of actin dots covering each side of the plaques was scored in resting, C-shaped, U-shaped and Instable plaques. More than 100 fields were counted for each experiment and statistical analysis was performed by ANOVA.

## Results

**Fig. 1:**
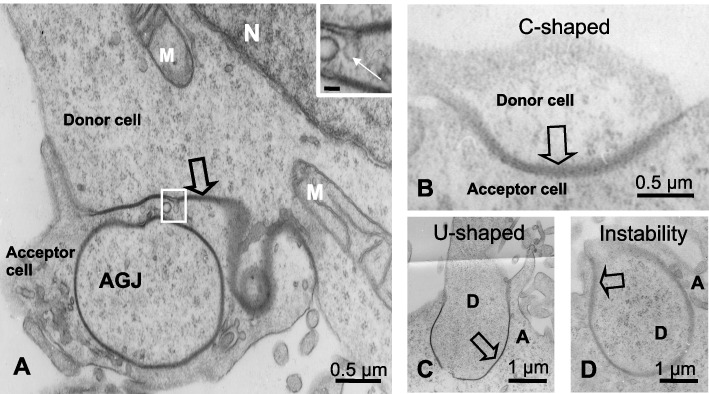
Gap junctions examined in cultured Cx43-GFP transfected Sertoli cells by electron microscopy. **A** This electron micrograph shows the junction between two cells, separated by an electron dense line corresponding to a GJP (large open arrow). The Donor cell contains large mitochondria (M) and part of the nucleus (N) is visible. A large AGJ is present in the cytoplasm of the Acceptor cell. The curved GJP originating from the Donor cell, is indicated by an arrow. The inset (Bar 50 nm) shows the area outlined by the white rectangle at higher magnification, showing a small endocytic vesicle (white arrow). **B** A curved C-shaped plaque (open arrow) separates the two adjacent Donor and Acceptor cells. **C** A U-shaped plaque (open arrow) defines the Donor (D) and the Acceptor (A) cells. **D** An Instability-shaped plaque (open arrow) precedes the future AGJ (Donor cell: D; Acceptor cell: A)

Ultrastructural studies made it possible to determine the different steps of GJP endocytosis in cultured Sertoli cells. Between the Donor (upper cell) and Acceptor cells (cell below) separated by the plaque (Fig. 1A**, **open arrow), the electron dense GJP invaginated to give rise to a large Annular Gap Junction (AGJ), different from the small classical endocytic vesicles (Fig. 1A, inset). GJP deformation (open arrows) was outlined at the Electron Microscopic level, revealing first the C-shaped structure (Fig. 1B), followed by a U-shaped (Fig. 1C) and then the Instable form before the AGJ (Fig. 1D). Time lapse analysis of Cx43-GFP expressing cells, in Light Microscopic images, depicted a similar progressive curvature of the Cx43-GFP labelled plaque (data not shown).

**Fig. 2:**
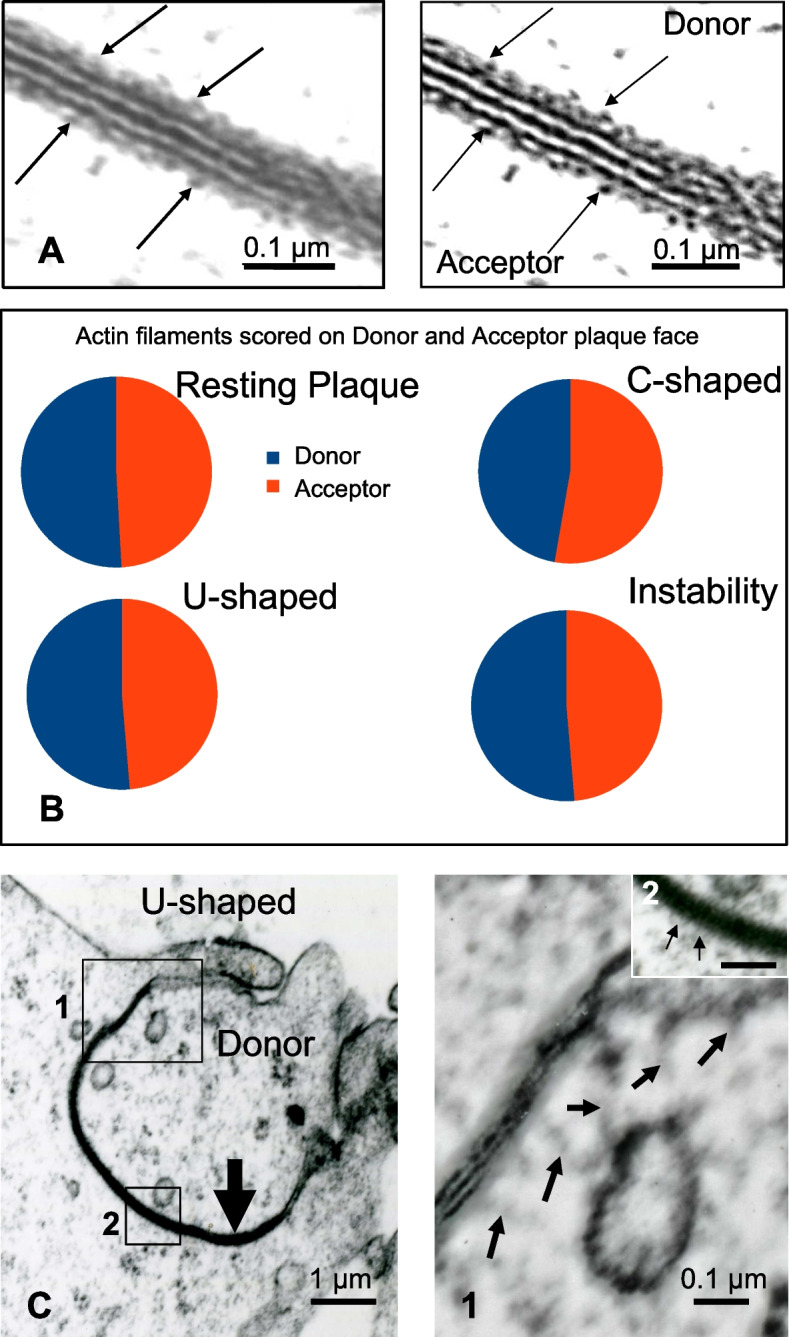
Electron microscopy analysis of actin organization during GJP endocytosis **A** Image in cross section of part of a GJP trilaminar structure reveals the peripheral irregular festoon of actin (left panel, arrows). ImageJ Enhanced Contrast clarifies the spiny structure of the actin decorating both sides of the GJP (right panel, arrows). **B** Semiquantitative analysis of actin-like spines decorating the GJP during the different steps of endocytosis before AGJ formation shows that the number of fine filaments present on Donor or Acceptor cells side is not significantly different. M**o**re than one hundred images in each cases. **C** Electron microscopy image of a U-Shaped plaque (left panel). Careful examination reveals that fine elongated actin filaments are oriented towards the cytoplasm and are mainly attached to the donor side of the GJP edge (right panel, inset 1, arrowheads), whereas numerous short actin filaments emanate from the acceptor side of the GJP (inset 2, arrows)

Since actin-depolymerization drugs are known to alter AGJ formation [11], we then explored the distribution of actin filaments at the GJP during the endocytic process by Electron microscopy (Fig. 2A, left panel). The Enhanced Contrast function of ImageJ made it possible to visualize actin filaments near the plasma membrane more precisely (Fig. 2A**, **right panel, arrows). The images revealed that actin cables were present in both sides of the straight GJP (Fig. 2A). Semi-quantitative analysis of actin-like spines decorating the GJP indicated that the number of actin filaments detected on Donor cells versus Acceptor cells was not significantly different during the progressive deformation of the GJP from C-shaped form to instability (Fig. 2B). However, further examination revealed that during GJP endocytosis, specific actin filament organization was noticed at the edges of the GJP, mostly on U-shaped form (Fig. 2C, left panel). In addition, the images revealed that the actin filaments decorating the Donor cell side were much longer (arrows) than any other actin spots located along the GJP (Fig. 2C, left and right panels), suggesting particular actin forces acting at the GJP edges.

**Fig. 3:**
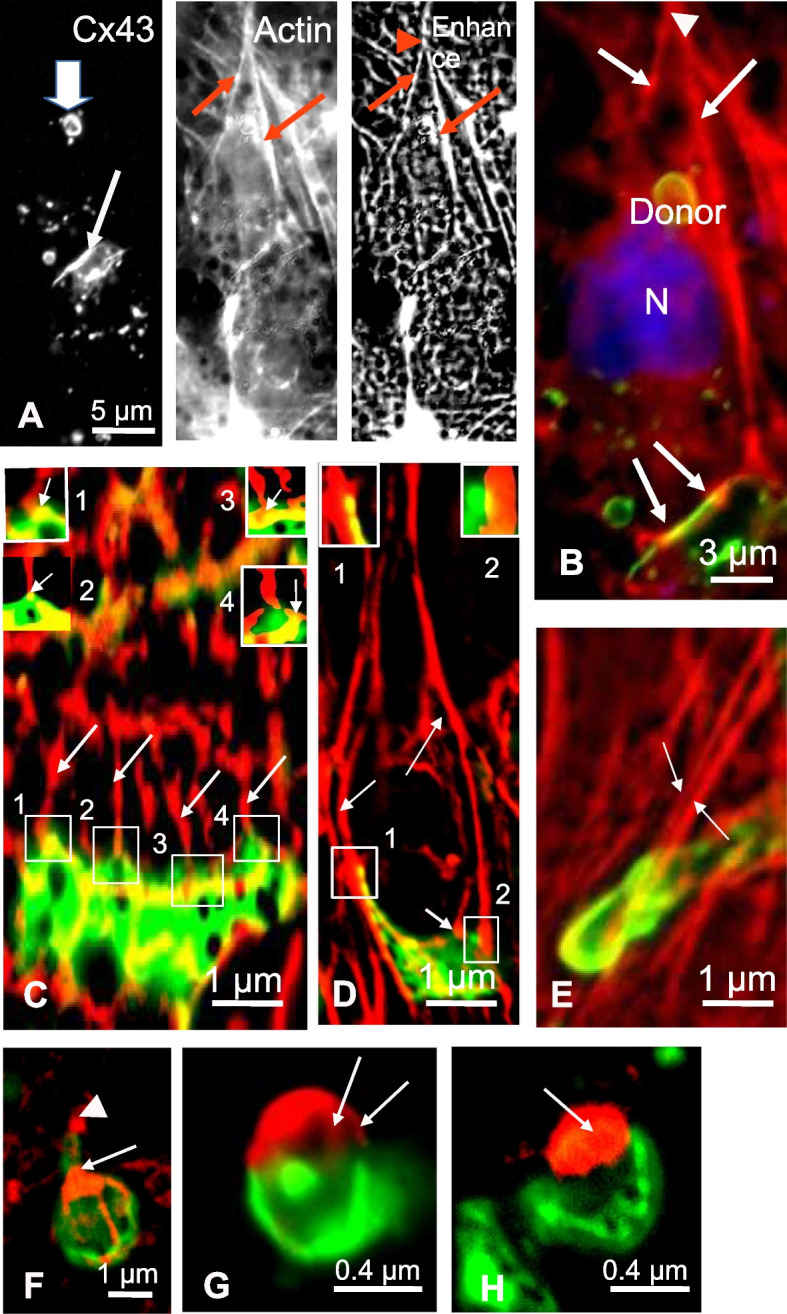
Deconvolution microscopy of GJP endocytosis in Cx43-GFP transfected cells and relation with actin cords. **A** A GJP (arrow), and an AGJ (large white arrow) are shown. High magnification of the actin distribution reveals the presence of long straight lines (middle panel, red arrows). ImageJ Enhanced local contrast of the same section confirms the organization of actin cables (right panel, red arrows) and shows that the elongated actin cables fuse at a single point far from the plaque (arrowhead). **B** Superimposition of GFP plaque (green) with actin fibers (red) highlights the specific attachment of actin cables on GJP edges (yellow spots, arrows). **C** A large GJP (green) seen in face view illustrates the numerous actin cables attached to this structure (arrows). The insets (1–4) point to the contact between single cables and the edge of the GJP (arrows on yellow spots). **D** In a C-shaped plaque (green), long actin cables (arrows, red) originating from its edges form long cables that approach from a distance far from the plaque. Three cables (arrows) are attached to the curved plaque and junction points are mainly seen at the extremities (yellow dots in the insets). **E** The U-shaped plaque (green) is prolonged by nearly parallel running actin cables (arrows). During the final steps of GJP endocytosis, from the U-shaped to the unstable form of the GJP, the actin structure changes drastically. **F, G** The actin cables seem to fuse in a single strand (arrowhead in F) and the remnant cables associated with the curved plaque remain visible; a large actin mass is observed (arrow). The remnant actin cables, detached from the opposite side of the plaque, come close to the future mouth of the forming AGJ and accumulate in a large actin mass (arrows). In G, fine residual actin cords remain attached to the plaque (arrows) on one side and fuse together on the actin mass. **H** Finally, the final actin mass is located over the mouth of the newly formed AGJ

We further explored the actin organization around the GJP during endocytosis by investigating the presence of GJP and AGJ in Cx43-GFP expressing cells (Fig. 3A, left panel). High resolution light microscopy in Cx43-GFP expressing cells, labeled with Phalloidin-coupled rhodamine to reveal actin, showed that actin filaments were distributed all along the cell periphery and sparsely contacted GJP (Fig. 3B, arrows). Higher magnification revealed that elongated actin cords fused at single points (Fig. 3A, middle panel), as better reflected in enhanced contrast images (Fig. 3A, right panel). Strikingly, closer look at the interactions between actin filaments and GJP by means of ImageJ Enhance Contrast software indicated that contacts occurred almost exclusively at the edges of the GJP, as unique points connecting perpendicular actin stress fibers to the Gap junction, and exclusively in the Donor cells (Fig. 3B, yellow spots). The number of actin cables connecting GJP at the beginning of the endocytic process was found to be about 3 at a minimum, increasing to a maximum of 9 for the largest plaques per GJP (Fig. 3C), and were located at the periphery of the curving plaque (Fig. 3D). The insets in Fig. 3C & D focus on the insertion of the cords at the plaque edges (yellow spots). The presence of these cables connecting the edge of the GJP was also confirmed by EM (Fig. 2C). Examination of the last steps of the GJP invagination from the U-form to the unstable form before AGJ formation suggested that the ultimate destination of the actin cables was to be reorganized as an actin mass close to the forming, round AGJ (Fig. 3H). First, the actin cables came closer to the others (Fig. 3E) and detached from the opposite face of the cell. Second, a large actin mass resulting from compaction of the actin cords was noticed close to the U and unstable shaped plaques (Fig. 3F, G and H). Third, remnant actin cables remained visible between actin mass and the forming AGJ (Fig. 3F and G).

**Fig. 4:**
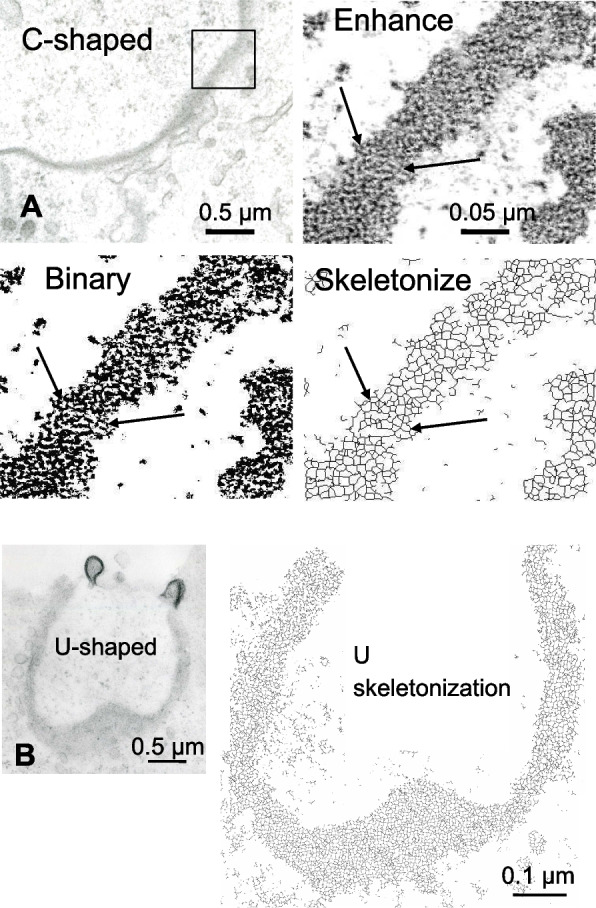
Actin organization in C-shaped and U-shaped plaques analysed at the ultrastructure level associated with enhanced local contrast and skeletonization. **A** Careful examination after enhanced contrast of a selected region of a C-shaped plaque (upper left panel), indicated in the inset (upper right panel), demonstrates the presence of fine anastomosed filaments structuring a large network (arrows). The binary 8 bit (lower left panel) and the final skeletonized images (lower right panel) of the same region highlight the presence of fine actin meshes composing the network (lower left and right panels, arrows). **B** Similar image manipulation of a U-shaped plaque confirms that the actin network covers the entire structure of the plaque (arrows)

To better visualize the fine actin network superimposed on plaque face, a C-shaped plaque was analyzed (Fig. 4A, upper left panel). The Enhance Contrast function of ImageJ used to examine a crop region of the plaque gave better contrast of the delicate linear structure covering the plaque (Fig. 4A, upper right panel). After image binarization (Fig. 4A, lower left panel) and skeletonization (Fig. 4A, lower right panel), the actin network was clearly shown.

Using similar filtering on the U-shaped plaque, seen from a partial face view, this actin network organization could be seen to be conserved throughout the endocytic process (Fig. 4B).

The fine and rapid modifications of plaque edges was better appreciated using Spinning disk confocal microscopy and time-lapse analysis (supplemental Figure). The spiny aspect of the edge of the U-shaped plaque seems modified. Actin skeletonization clearly demonstrated the actin cable attachment at the periphery of the GJP (supplemental Figure, arrows).

**Fig. 5:**
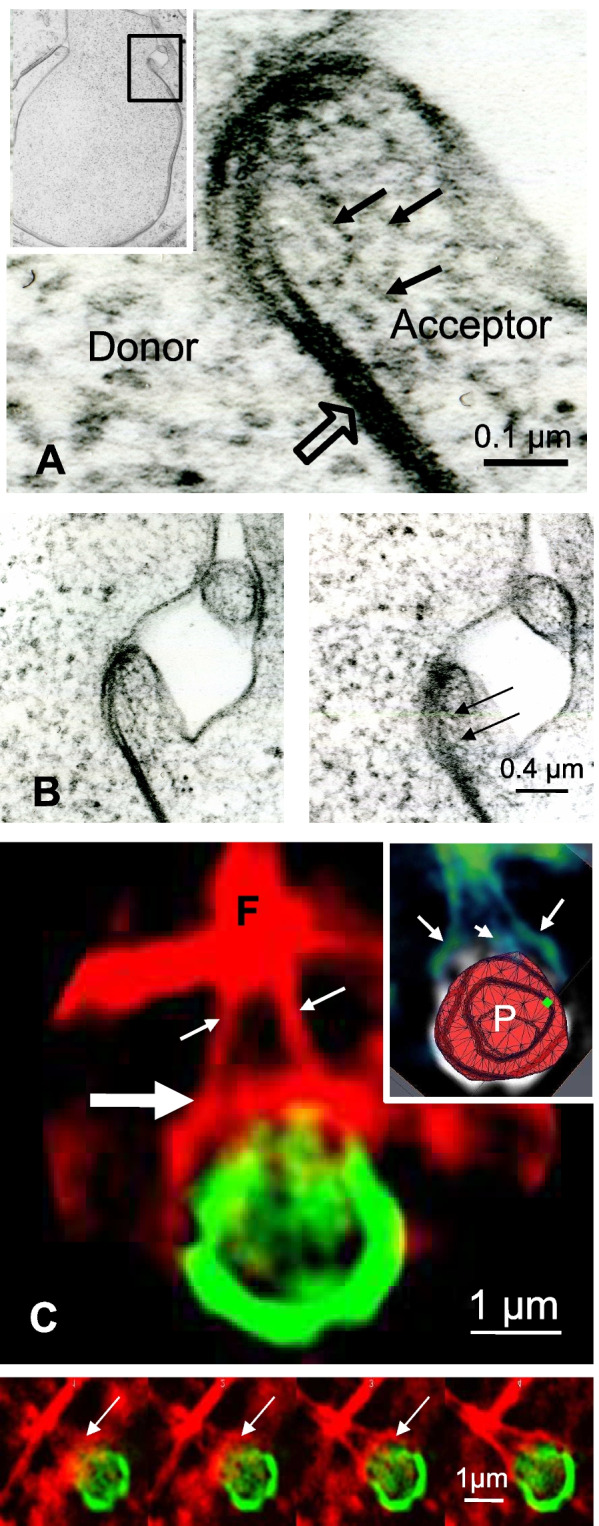
Remodeling of actin organization during the last steps of GJP endocytosis. **A** High magnification of part of a U-shaped plaque (inset) shows that the cytoplasmic fold of the Acceptor cell that encloses the GJP mouth contains numerous actin filaments more or less elongated (arrows). Actin filaments are more condensed in this particular region. **B** Pair of stereoscopic images, examined with a stereoscope, reveals the 3D organization of the actin filaments present in the small membranous region delimited by the Acceptor membrane. The fine spatial examination of actin distribution reveals that most actin filaments ran parallel to the others and that they are also directed parallel to the plasma membrane (arrows in right panel). **C** In Cx43-GFP transfected Sertoli cells, the U-shaped round plaque (green) is prolonged by two red actin cables (white arrows). Their fusion point (F) is located close to the plaque. A ring-like structure formed by actin filaments is close to the mouth region of the GJP (large white arrow). 3D reconstruction of the GJP (inset) clearly indicates actin cables (arrows) inserted on the periphery of the plaque (P). Confirmation of the presence of an actin ring that embraces the region of the final closure of the GJP is given by analysis of Z series of this image (lower panels in C, arrows)

Ultrastructural examination of the edge of a U-shaped plaque showed that a membranous folding started from the trilaminar membrane of the plaque in the Acceptor cell on one side and in the Donor cell on the opposite face (Fig. 5A, inset). In the narrow cytoplasmic region delimited by the plasma membrane of the Acceptor cell, numerous thin anastomosed filaments of resembling actin appeared (Fig. 5A, small arrows). This filamentous accumulation progressively disappeared with increasing distance from the membrane folding. EM stereo images showed that numerous elongated filaments ran parallel to the cytoplasmic plasma membrane (Fig. 5B**,** arrows), revealing a cluster of cytoskeletal molecules, connected with small, short anastomotic filaments.

In order to better understand the complete 3D organization of actin in these regions, immunofluorescence experiments on Cx43-GFP expressing cells associated with Rhodamine labelling for actin were performed. In a U-shaped plaque (Fig. 5C, green), two actin cables originating from their fusion point (**F**) connected the GJP edges (Fig. 5C, small arrows). Standing out particularly in the mouth region of the plaque, continuous actin cables seemed to embrace this region (Fig. 5C, large arrow). Z series from the 3D immunofluorescence experiments of the U-shaped plaque demonstrated that this round actin structure surrounded the GJP opening (Fig. 5C). EM stereo images showed that this actin ring-like structure, which was only observed during the last steps of GJP endocytosis, originated from the Acceptor cell (Fig. 5B).

## Discussion

Gap junction plaque (GJP) endocytosis and formation of the AGJ is a complex process initiated by both a Donor and an Acceptor cell, leading to the formation of the future annular structure. This ‘nomenclature’ has been used by several authors to specifically detail the involvement of cells forming functional GJ [28]. The present investigation demonstrates for the first time that two adjacent cells can collaborate in internalizing the giant membranous GJP and that the actin network from both cells is actively involved in this specific endocytic process. High resolution images have made it possible to describe the successive steps of this sort of cellular physiological process.

Previous studies demonstrated the existence of strong interactions between Cx, the only component of GJP, and many proteins partners including actin [29]. Although the involvement of actin in GJ formation and stabilization has been well documented [15, 30], its implication during the different steps of GJP endocytosis remains unclear. Previous works have suggested that actin may also participate in GJP endocytosis [19, 20], however, the mechanisms by which actin participates in such processes have not been clearly elucidated.

It is now well established that actin filaments assemble into diverse structures to provide forces for several vital cellular processes. During small endocytic vesicle internalization, actin traction has been demonstrated [22]. However other authors studying the mechanical forces exerted by actin to internalize the large GJP, as compared to small vesicles, considered that actin forces are probably not sufficient to curve the large trilaminar membranous structure to allow complete internalization [31]. In the present investigation, Dmitrief (personal communication) attempted such calculation, demonstrating that strong forces are indeed necessary to obtain plaque curvature. Thus, we questioned how GJP, which are much larger structures than classical small endocytic vesicles, can find the forces that pull them deep into the Acceptor cells, and what is the role of actin?

In the present work, stereoscopic examination of EM contrasted images, performed to precisely examine actin location, revealed that fine actin filaments are organized as a continuous network superimposed on the GJP surface. Such an actin network located close to the cortical membranous region has been recently quantified by super resolution microscopy [32]. However, quantitative analysis of actin filaments over both the face of the GJP on Donor and that on Acceptor gives no precise information on the possible reorganization of actin filaments during internalization. By combining 3D EM and LM observations at high resolution, we have been able to report, for the first time, the presence of elongated actin filaments originating from the Donor cell located at the periphery of the GJP and associated with the actin network linked to the GJP. These observations are supported by time-lapse spinning disk microscopy examination of Cx43-GFP expressing transfected cells that pointed out in real time the peripheral modifications of the plaque edges at the level of the attachment points between actin cables and the GJP (see supplemental figure). This original approach has made it possible to visualize in real time the rapid and fine modifications of the plaque edges under traction by actin cables. The GJP edges appeared festooned and their delicate remodeling clearly confirms the traction force applied by the actin cables.

Thus, the physical forces exerted by peripherally located actin cables could transmit traction forces to the entire actin network covering the plaque, resulting in GJP deformation. This is strongly supported by previous data in oligodendrocytes showing that the actin network is deformed by traction of actin cables [33] and that cortical actin cables play a key role in cell curvature in yeast [34]. Additionally, acto-myosin forces forming stress fibers can affect cell shape [35]. More recently, the possibility that actin stress fiber geometry governs the forces and acts directly over the cytoskeletal network has been suggested [36]. Other studies demonstrated that mechanical forces mediated by actin can modify the branched actin network during dynamic lamellipodial protrusions [37]. Thus, one may speculate here that the peripheral actin cables transmit forces over the fine actin network covering the GJP allowing traction of the plaque edges to initiate the C-shaped modulation of the GJP. In addition, the current observations suggest that just a few peripheral actin cables (at least three) would be sufficient to transmit enough force to the overall fine actin network covering the plaque surface to initiate the curvature of the entire trilamellar membranous structure.

Here, we have shown that actin cables are permanently attached to the plaque edges throughout the endocytic GJP progress, from the C-shaped form to instability. We have also demonstrated that at the opposite side of the plaque, the actin cables issuing from the GJP fused to form an actin fusion point corresponding to a focal adhesion. Focal adhesion has been described in many cell types as the result of cytoskeletal component assembly [38]. Such a fused region of actin cables has been also described in cultured epithelial cells and fibroblasts [39]. From the U-shaped plaque to instability, surprisingly, the fusion point seemed to detach from the plasma membrane and became a large actin mass near the future closure region of the forming AGJ (Fig. 5), as observed in the current immunofluorescence experiments. Subsequent EM and LM studies also revealed that remnant short actin cables are still visible during this dynamic process, in contrast to the larger actin mass formed. Such an actin mass is probably the result of actin cable disorganization during GJP endocytosis.

During the last step of GJP endocytosis, IF and 3D EM images revealed that actin filaments were mainly present as an actin ring-like structure located in the cytoplasmic fold delimited by the Acceptor cell membrane in continuity with the plaque edges. Such a specific actin structure, which has yet been observed in endothelial cells [40], could result from remodeling of cortical actin [41]. In addition, recent results have reported that actin stress fibers can assemble directly from the cortical actin meshwork [42]. Together, these data support the hypothesis that the actin ring observed during the last steps of GJP endocytosis originates from the actin filaments still present in the specific cytoplasmic region of the Acceptor cell that embraces the Donor cell. We thus propose that the forces developed by the actin ring in this specific cytoplasmic region are necessary to complete the GJC endocytic process. Interestingly, other studies have reported that an actomyosin ring formation could help the final exocytosis of large granules [40, 43]. Thus, forces exerted by this particular actin structure would allow completion of the endocytic process of the large GJP structure into AGJ, suggesting that the actin ring could be involved not only in exocytosis but also in endocytosis. While these data have clarified the role of actin in GJP endocytosis, they do not, however, exclude that in addition to actin, other Cx protein partners previously reported may also participate in GJP endocytosis.

## Conclusion

The present investigation demonstrates that GJP endocytosis requires close cooperation between two adjacent cells. First, the Donor cell initiates the curvature of the plaque, but could probably not finalize the complete AGJ formation due to the large forces necessary for such function. The progressive reorganization of actin cables finally results in the formation of a large non-functional actin mass. Second, the Acceptor cell, appears to be involved through the formation of an annular sub-membrane actin ring that surrounds the opening of the closure region of the U-shaped plaque, as for exocytic large membranous granules [40, 43]. We conclude that this dynamic actin ring structure in the Acceptor cell is thus able to produce enough force to close the instable plaque and to finalize GJP endocytosis initiated by the Donor cell.

## Supplementary Information

**Additional file 1: Supplemental Figure.** Spinningdisk examination of actin cables dynamic versus GJP movment. (**A**) Examination of the dynamic distribution of the red actin cables (white arrows) over a U-shaped plaque (green, black arrow). Rapid time-lapse video microscopy reveals the festooned aspect of the GJP edges (black arrows). The spines formed by the contour of the GJP edges change shape with time. Skeletonization of actin cables using ImageJ show precise contour of some cables attached to plaque edges. The cables show clear modifications in their organization but remain inserted in the GJP throughout endocytosis. (**B**) ImageJ skeletonization after enhanced contrast clearly demonstrated the fine actin cables (red arrows). Each plaque spine appeared to be connected to the actin cables.

## Data Availability

Yes.

## Abbreviations

AGJ: Annular Gap Junction
GJ: Gap Junction
GJP: Gap Junction Plaque
Cx43: Connexin43
GFP: Green Fluorescence
EM: Electron microscopy
LM: Light microscopy
TIRF: Total Internal Reflection Fluorescence
