# Supplementary figures and images for: Cooperative cell–cell actin network remodeling to perform Gap junction endocytosis
Source: Basic Clin Androl. 2023 Aug 3;33:20. doi: 10.1186/s12610-023-00194-y (PMC10399049; doi:10.1186/s12610-023-00194-y)

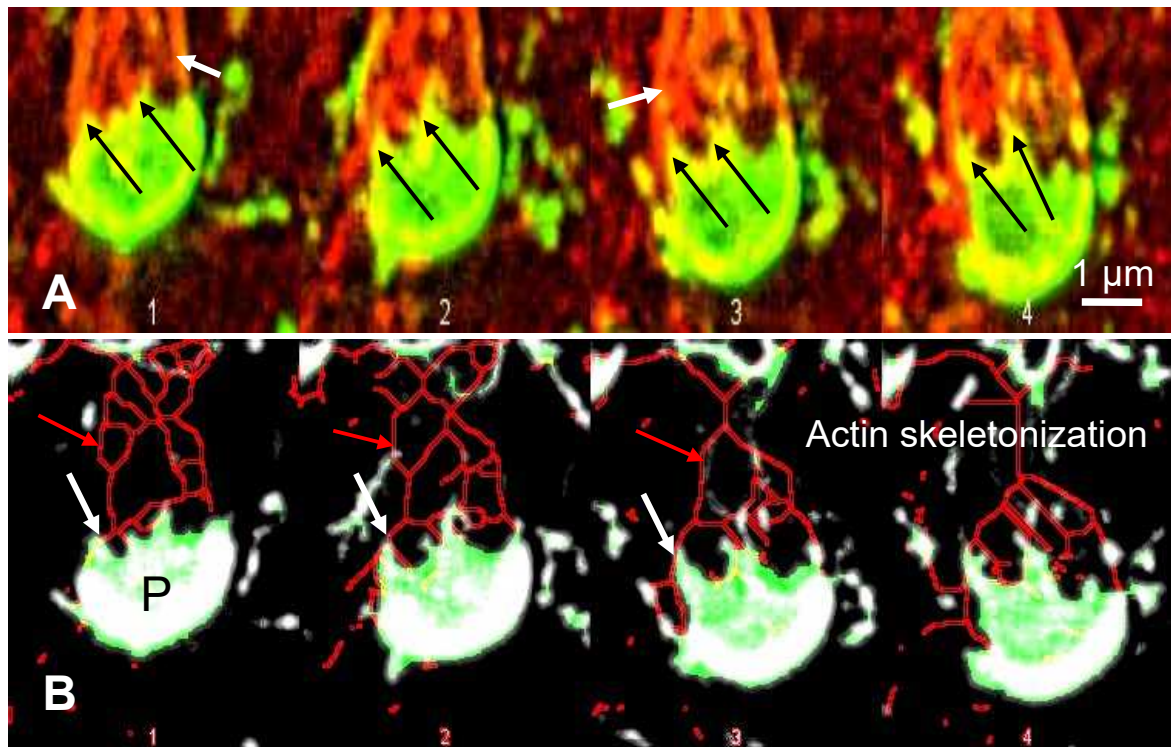

Supplemental Figure, Segretain D et al 2022

Supplement: Supplementary file 1 — Additional file 1: Supplemental Figure. Spinningdisk examination of actin cables dynamic versus GJP movment. (A) Examination of the dynamic distribution of the red actin cables (white arrows) over a U-shaped plaque (green, black arrow). Rapid time-lapse video microscopy reveals the festooned aspect of the GJP edges (black arrows). The spines formed by the contour of the GJP edges change shape with time. Skeletonization of actin cables using ImageJ show precise contour of some cables attached to plaque edges. The cables show clear modifications in their organization but remain inserted in the GJP throughout endocytosis. (B) ImageJ skeletonization after enhanced contrast clearly demonstrated the fine actin cables (red arrows). Each plaque spine appeared to be connected to the actin cables. [file 12610_2023_194_MOESM1_ESM.pdf]
